# Risk of transmission via medical employees and importance of routine infection-prevention policy in a nosocomial outbreak of Middle East respiratory syndrome (MERS): a descriptive analysis from a tertiary care hospital in South Korea

**DOI:** 10.1186/s12890-019-0940-5

**Published:** 2019-10-30

**Authors:** Hyun Kyun Ki, Sang Kuk Han, Jun Seong Son, Sang O Park

**Affiliations:** 1 Division of infectious diseases, Department of Internal Medicine, School of Medicine, Konkuk University, Konkuk University Medical Centre, 120-1 Neungdong-ro (Hwayang-dong), Gwangjin-gu, Seoul, 05029 Republic of Korea; 2 Department of Emergency Medicine, Kangbuk Samsung Hospital, Sungkyunkwan University School of Medicine, 108 Pyung-Dong, Jongno-Gu, Seoul, 110-746 Republic of Korea; 3 Division of Infectious Diseases, Department of Internal Medicine, Kyung Hee University Hospital at Gangdong, 892, Dongnam-ro, Gangdong-gu, Seoul, Republic of Korea; 4 Department of Emergency Medicine, School of Medicine, Konkuk University, Konkuk University Medical Centre, 120-1 Neungdong-ro (Hwayang-dong), Gwangjin-gu, Seoul, 05029 Republic of Korea

**Keywords:** Middle East respiratory syndrome coronavirus, Nosocomial infection, Infection control, Isolation, Hand hygiene

## Abstract

**Background:**

In 2015, South Korea experienced an outbreak of Middle East respiratory syndrome (MERS), and our hospital experienced a nosocomial MERS infection. We performed a comprehensive analysis to identify the MERS transmission route and the ability of our routine infection-prevention policy to control this outbreak.

**Methods:**

This is a case–cohort study of retrospectively analysed data from medical charts, closed-circuit television, personal interviews and a national database. We analysed data of people at risk of MERS transmission including 228 in the emergency department (ED) and 218 in general wards (GW). Data of personnel location and movement, personal protection equipment and hand hygiene was recorded. Transmission risk was determined as the extent of exposure to the index patient: 1) high risk: staying within 2 m; 2) intermediate risk: staying in the same room at same time; and 3) low risk: only staying in the same department without contact.

**Results:**

The index patient was an old patient admitted to our hospital. 11 transmissions from the index patient were identified; 4 were infected in our hospital. Personnel in the ED exhibited higher rates of compliance with routine infection-prevention methods as observed objectively: 93% wore a surgical mask and 95.6% washed their hands. Only 1.8% of personnel were observed to wear a surgical mask in the GW. ED had a higher percentage of high-risk individuals compared with the GW (14.5% vs. 2.8%), but the attack rate was higher in the GW (16.7%; l/6) than in the ED (3%; 1/33). There were no transmissions in the intermediate- and low-risk groups in the ED. Otherwise 2 patients were infected in the GW among the low-risk group. MERS were transmitted to them indirectly by staff who cared for the index patient.

**Conclusions:**

Our study provide compelling evidence that routine infection-prevention policies can greatly reduce nosocomial transmission of MERS. Conventional isolation is established mainly from contact tracing of patients during a MERS outbreak. But it should be extended to all people treated by any medical employee who has contact with MERS patients.

**Trial registration:**

NCT02605109, date of registration: 11th November 2015.

## Background

Middle East respiratory syndrome (MERS) is a respiratory disease caused by a novel single-stranded beta-coronavirus, which was first reported in patients residing in Saudi Arabia in 2012 [1]. The primary transmission was thought to be from zoonotic exposure in this area [2]. However, some clusters of outbreaks from secondary transmission in the health care setting have been reported, and human-to-human transmission is considered to play an important role in secondary transmission [3–5]. Until May 2015, outbreaks of MERS were mainly restricted to countries in the Middle East including Saudi Arabia, Jordan, Kuwait, Lebanon and Qatar [6]. Regions outside the Middle East, such as Europe, USA and some Asian countries have not generally experienced noticeable outbreaks of this virus [7–10].

In 2015, South Korea experienced the largest outbreak of MERS outside the Middle East. A total of 186 patients were serologically confirmed as having MERS between May and July 2015 in Korea [11]. Most of these MERS infections were identified as having arisen from human-to-human transmission in the health care setting [12]. Given that the emergency department (ED) plays an important role in providing the main care for acutely ill patients, the ED can be an open portal for transmission of pathogens into a hospital system. Unrecognized patient who visited in the ED greatly contributed to wide spreading of MERS for less time [13].

**Fig. 1:**
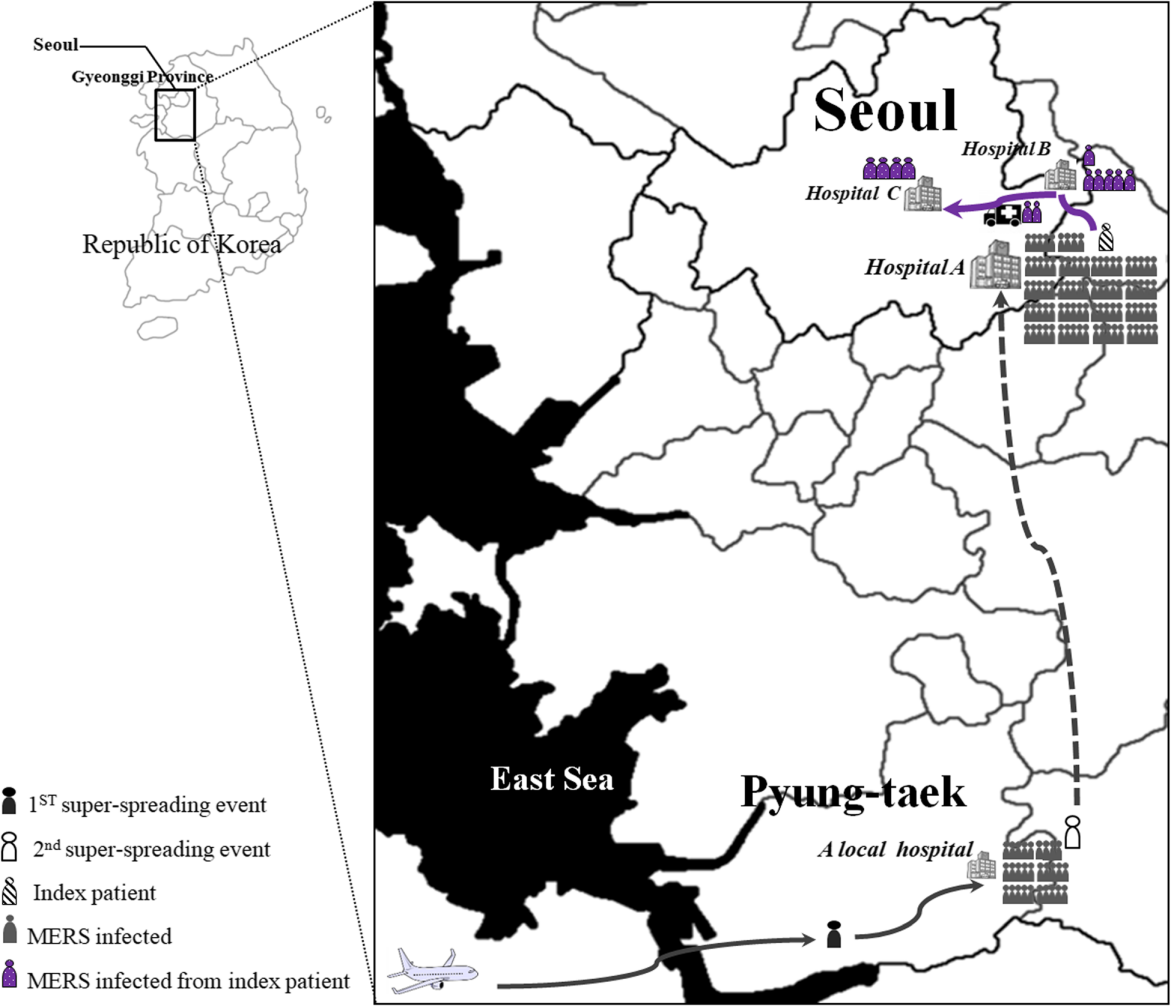
Early transmission tree of Middle East related to the index patient. (This figure was redrawn by authors using the free map samples which do not require copyright permission)

The first human-to-human transmission of MERS in South Korea occurred in an old Korean business man who travelled to the Middle East area in May 2016 (Fig. 1) [12]. He visited a local clinic in Pyung-Taek because of a high fever and coughing. To provide care for pneumonia, he was admitted to the GW in a local hospital in Pyung-Taek in Gyeonggi Province. During the admission, 28 people who had been admitted to this GW in this hospital were infected (1st super-spreading event of the Korean Outbreak), and some of these people moved to other tertiary hospitals by themselves. Among them, a young male patient visited the ED of Hospital A in Seoul on 27 May (Fig. 1). He complained of severe fever, coughing and sputum and treated during two days. MERS was confirmed on 29 May, and a total of 90 transmissions were identified in Hospital A (2nd super-spreading event) [14]. An unrecognized patient who was infected in Hospital A visited in Hospital B and C, and numerous people were exposed directly or indirectly to this patient in the hospital setting.

After outbreak, we performed a post-outbreak analysis with detailed observational data from our hospital (Hospital C). Our report may be helpful for further understanding of MERS transmission and the role of routine infection-prevention policies in reducing nosocomial outbreaks of MERS in health-care settings.

## Methods

### Overview of the study and setting

This was a case–cohort study of retrospectively analysed experiences of an in-hospital MERS outbreak in Hospital C in South Korea. The study protocol was reviewed and approved by the Institutional Review Board of Hospital C. According to the study protocol, anonymous clinical or public data was used without the need for consent in the interest of public health. We obtained verbal consent from participants to obtain personal information from a closed-loop interview or review of recorded closed-loop video data in the hospital. Other public data about MERS in Korea were obtained from the open data available from an online MERS communicating system or the Korean health Care Database.

Our hospital (Hospital C) is an 870-bed university hospital located in the north-east of Seoul, South Korea. Annually, > 50,000 patients visit our ED. The ED is located on the first floor of the main hospital building and has 5 treatment zones: 1 zone containing 4 beds for intensive care, 2 large rooms each containing 10 beds, and one paediatric care room containing 10 beds. For fast radiological evaluation of ED patients, a radiology suite and a computed tomography suite are located adjacent to the ED. The hospital has 7 floors for in-patient care, each of which contains 2 general wards (GWs) for admitted patients. Each GW comprises 10 rooms with 6 beds each and 7 rooms with 1 or 2 beds each.

### Hospital prevention policy and measures during the pre- and post-isolation periods

Before the MERS index patient visited our hospital, we had strengthened the triage system for identifying suspected MERS patients. We set up the MERS triage room outside of the ED. For patients complaining of any symptoms of MERS, including fever, cough, sore throat, rhinorrhoea, shortness of breath, nausea, vomiting or diarrhoea, triage staffs routinely checked the histories of exposure to confirmed MERS cases. Triage staffs also checked for recent travel within the Middle East area or visits to hospitals or local clinics where MERS cases had been diagnosed during the last two weeks. All suspected MERS patients were not permitted to remain in the ED, they were moved directly to the temporary isolation room outside of the ED. Given the increasing risk of unrecognized infective patients visiting our hospital, we encouraged all healthcare providers (HCPs) to wear surgical mask routinely and gloves during procedures and to use proper hand hygiene in the ED and outpatient department. Our hospital also provides a surgical mask routinely to all patients and visitors who stay in the ED, and we encourage frequent hand washing by placing numerous hand sanitizers at all ED stations and beds.

After identification of the index patient, we categorized the transmission risk of all possible people (primary interest group) who were suspected of having had contact with the index patient or who had been treated in the same area in the ED or GW during the index patient’s stay. All people in the primary interest group were isolated for 14 days according to the national infection control guidelines of the Korean Ministry of Health if they had been in contact with the patient or objects touched by the patient (e.g. bed, blanket or other items) or had stayed within 2 m of the index patient [15].

Other people who had not been quarantined were categorized as the active surveillance group and were monitored daily by our hospital or a community health centre. During the 2–3 weeks of close observation, respiratory samples from sputum, naso- or oropharyngeal swabs were acquired for anyone in the isolated group or active surveillance group who displayed fever or any respiratory symptoms. The samples were sent to Korean national laboratory centres. MERS was confirmed in respiratory samples showing a positive result in a real-time reverse transcriptase polymerase chain reaction assay. If the initial test was negative, an additional assay was conducted using another respiratory sample [15].

### Post hoc analysis of the transmission tree, personal protection equipment (PPE) and hand hygiene

Post hoc analysis was performed after the end of the MERS outbreak in our hospital. The analysis focused mainly on data for the index patient and all people treated in the ED and GW during the pre-isolation period of the index patient. First, by reviewing the closed-circuit television (CCTV) and electronic medical charts, we traced the location and movements of the index patient and drew possible lines and zones of transmission risk. Second, we checked the direct transmission trees and contact events. For further analysis, we categorized all persons of interest into three groups according to their location and contact with the index patient as follows [14]. 1) The high-risk group was defined as patients who had been within about 2 m of the index patient. 2) The intermediate-risk group was defined as patients who had stayed in the same room as, but who had had no direct contact with, the index patient. 3) The low-risk group was defined as patients who had been treated on the same floor or department area but who had not shared a room with the index patient.

We also collected data about exposure time, duration of contact and suspected route of contact. We also checked PPE data including wearing of masks, gloves, preventive gowns or eye protection and hand hygiene (hand washing) for each person. All data were collected by personal interview and review of hospital CCTV, electronic medical records, epidemiological reports from the local community health centre and data reported by the Korean Centres for Disease Control and Prevention.

For statistical analysis, we used IBM SPSS statistical software package 23.0 (IBM Corp., Armonk, NJ). Variables were compared between two groups using an independent two-sample *t* test or Fisher’s exact test. Two-sided *p-*values < 0.05 were considered to be significant.

## Results

### Index patient

The index patient in our hospital was an old patient who was transported to our ED on 6 June for surgery for a fractured femur. This patient had received treatment for malignancy disease in the ED of Hospital A between 27 and 28 May. The 2nd super-spreading event of MERS occurred in Hospital A during the same time (Fig. 1).

**Fig. 2:**
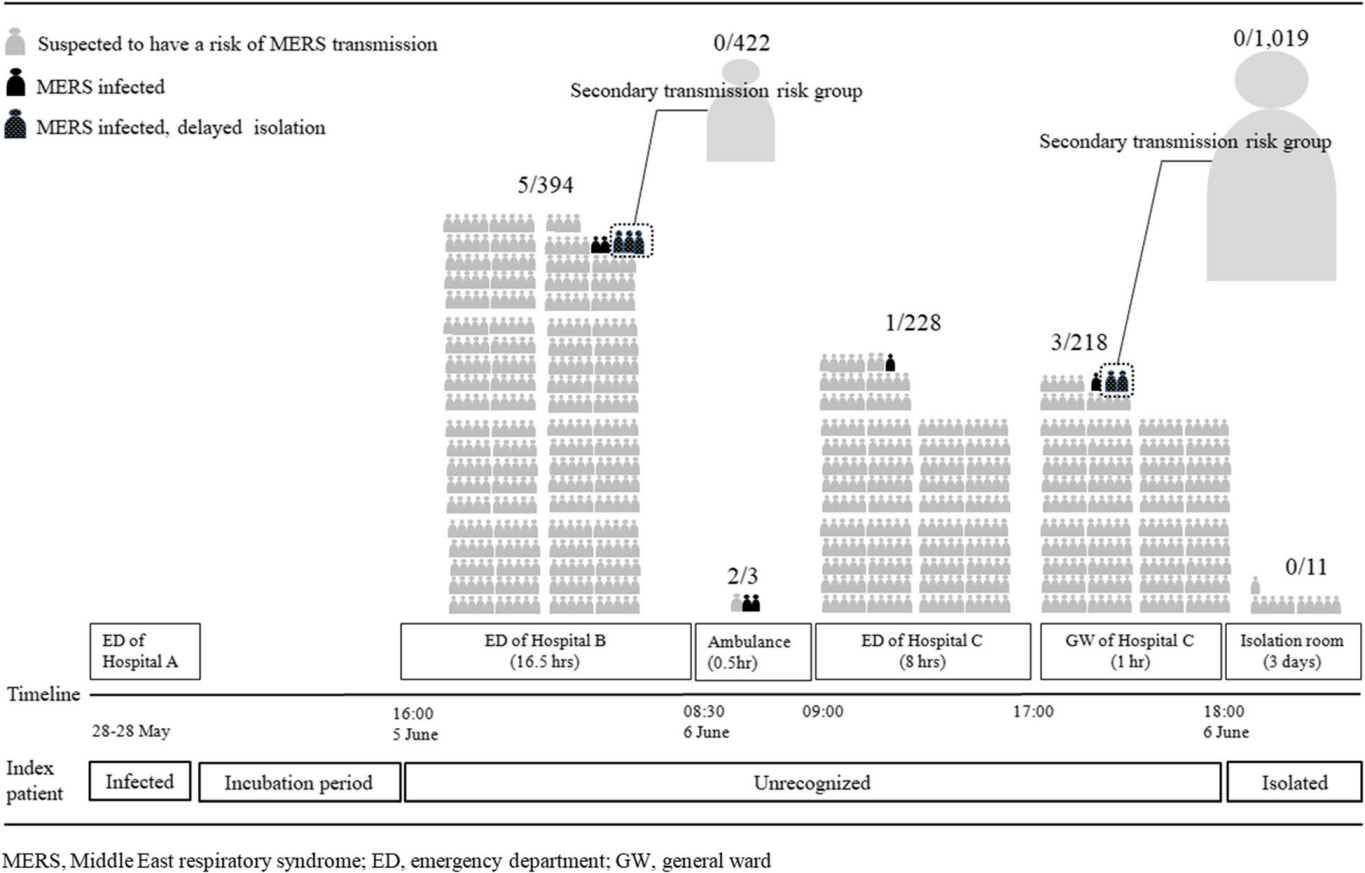
Timeline transmission tree of all of MERS patients infected by the index patient

After discharge from the ED of Hospital A, the index patient was transported to a local primary care facility and was admitted to the long-term care room for supportive care for 10 days (28 May to 5 June). This patient fell during this stay and was diagnosed with a left inter-trochanteric fracture on 5 June. The index patient was transferred to the ED of a nearby Hospital B for surgery on the inter-trochanteric fracture. The next day (6 May), this patient was transported to our ED at 9:00 am using a personal medical ambulance service at the request of the patient’s family (Fig. 2). On arrival at the ED, the patient had a slightly elevated body temperature of 37.5 °C, blood pressure of 136/56 mmHg, pulse rate of 76 beats/min and respiratory rate of ≈16 breaths/min. This patient did not exhibit fever, chilling, dyspnoea, coughing or productive sputum. This patient and caregivers denied any possibility of contact with MERS patients.

**Fig. 3:**
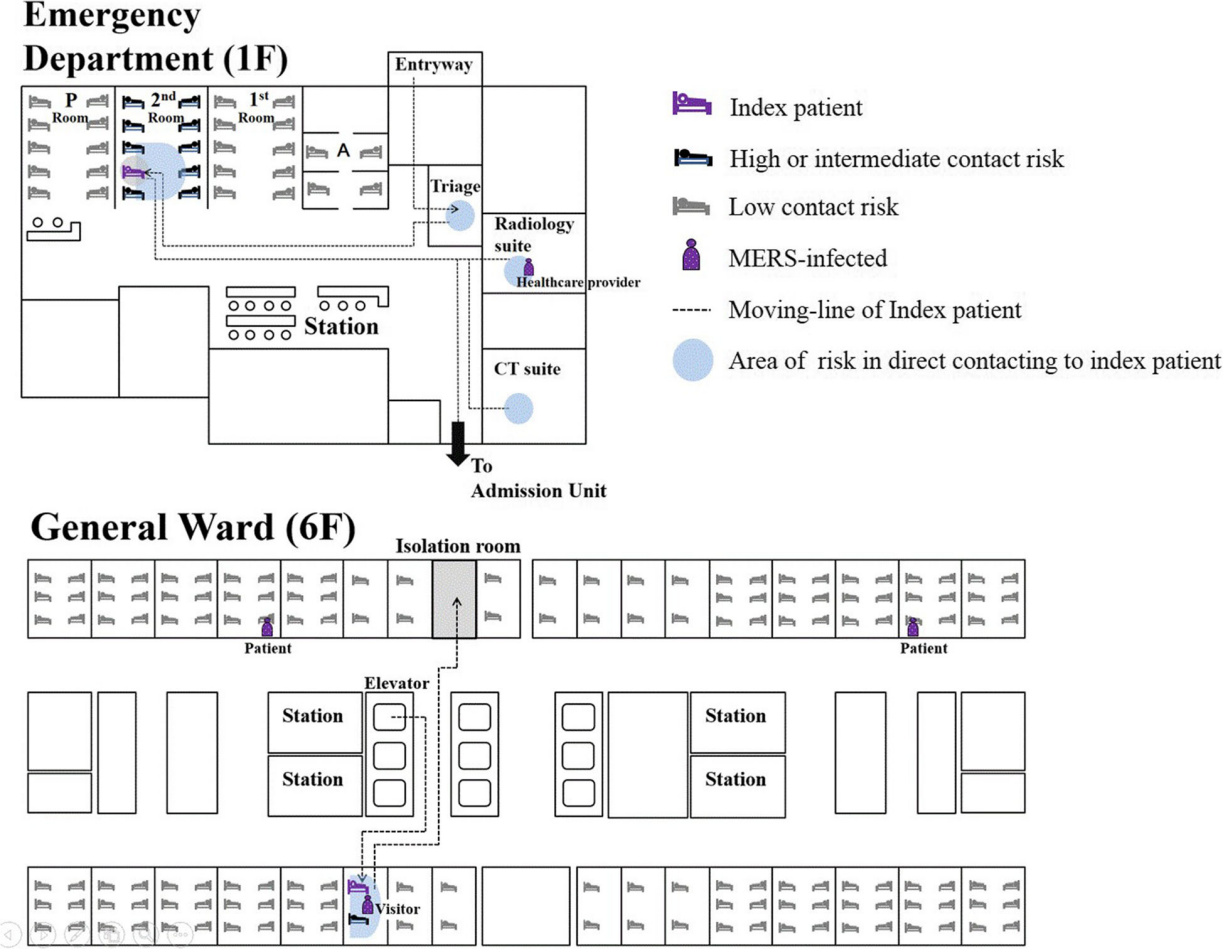
Schematic diagram for moving line of index patient and contact risk to other peoples during an 8-h stay on the emergency department (left-upper) and a 1-h stay on the general ward (lower)

Routine examination with chest X-ray in the ED showed suspicious pneumonic consolidation in the left lower lobe. For the elective operation to repair the femur fracture, the patient was admitted to the orthopaedic GW in our hospital at 5:00 pm. The index patient was first admitted to a two-bed hospital room but was then transferred to a single-bed hospital room after five minutes because the caregiver of the other patient staying in this room did not want to share the room with the index patient (Fig. 3).

High fever (39.1 °C) was measured in the initial vital sign check at 5:30 pm, and our hospital infection control unit was notified about the patient. The patient was evaluated further and her history taken again. This second evaluation revealed that the index patient had stayed in the ED of Hospital A, and this was confirmed by phoning Hospital A. The patient was then transferred to a negative-pressure isolation room according to the infection control guideline in our hospital. Her respiratory samples were sent to the Korean National laboratory centre, which confirmed a positive test result for MERS early the next day.

### Post-infection measurements and events after isolation of the index patient

After confirmation of MERS infection in the index patient, we could not identify those who had never had contact with the index patient or the virus-contaminated environment in the ED because of the frequencies of their movements within and around the ED and crowding. Therefore, all 228 people who had stayed in the ED were included as isolation subjects regardless of their time or position when in the ED. A total of 60 people who were suspected of contact with patients (high and intermediate risk group) were isolated at the hospital and 168 (low risk group) were isolated at their home. Otherwise, only 8 people (high and intermediate risk group) in the GW who had been in contact or shared a room with the index patient were isolated, because the index patient had stayed in the GW for only 1 h and her movements were limited. Other 210 people were not isolated because they had not been in contact with the index patient or virus-contaminated environment. Because of concern about indirect transmission by contact through virus shedding, we immediately performed environmental cleaning using bleach and alcohol of all possible areas in which the index patient had stayed or moved.

Along with the isolations of people and cleaning the environment after identification of the index patient, our infection control team added some new control measures. First, we campaigned for inpatients, hospital employees, and visitors to implement basic infection control including hand washing and wearing surgical masks. Second, because some missed cases can transmit MERS to other people before isolation is initiated, we performed routine daily checks of body temperature and monitored the onset of new symptoms for all inpatients and hospital employees.

### MERS transmission from the index patient

Table 1 summarizes the data for the 11 MERS patients who were infected by the index patient. Before the index patient visited our hospital, five (first to fifth patients in Table 1) were identified as having exhibited MERS in the ED of Hospital B. Two ambulance paramedics (Sixth and seventh patients) who had transported the index patient from Hospital B to our hospital were also infected with MERS (Table 1 and Fig. 2). They had briefly touched the index patient: one had briefly touched her to help her during the ride in the ambulance, and the other had only observed and cared for the patient during the 30 min of transport. Neither wore a surgical mask during transportation or when they entered our ED. Their recall of hand hygiene was unclear when questioned as part of this study.

**Table 1:** Summary of Middle East respiratory syndrome patients infected from index patient

Site	Index patient	No.	Position	Incubation period	Risk of contact	Result
Hospital B (6–7 June)	ED stay during 17 h	1	Patient	13 days	Unknown	Survived
		2	Patient	13 days	Unknown	Survived
		3	Care giver	16 days	High	Death
		4	Nurse	20 days	High	Survived
		5	Doctor	10 days	High	Survived
Transfer (7 June)	Ambulance during 10 min	6	Driver	6 days	High	Death
		7	EMT	7 days	High	Survived
Hospital C (7 June)	ED stay during 9 h	8	Radiologist	14 days	High	Survived
	GW stay during 1 h	9	Visitor	8 days	High	Survived
		10	Patient	15 days	Low	Survived
		11	Patient	17 days	Low	Survived

*ED* emergency department, *EMT* emergency medicine technician, *GW* general ward

During the index patient’s 8 h stay in the ED, MERS transmission within the ED space was not reported. Among the 228 isolated people, only 1 radiologist (eighth patient) who performed the X-ray of the index patient while wearing a radiology suit was diagnosed with MERS (Fig. 3). He had briefly touched the index patient (for < 5 min) while taking some simple X-ray.

Three people at GW were infected with MERS during the index patient’s 1-h stay in the GW. The first was a visitor (ninth patient) who stood briefly (1–2 min) beside the bed of the index patient. None of the other 7 of the isolated people who had been in the GW at this time were infected. The other 2 MERS patients (tenth and eleventh patients) were identified among a group of people who were not initially isolated. One (tenth patient) had been admitted in the same ward area and the other (eleventh patient) had been admitted in another ward on the same floor (Fig. 3). There was no contact or overlap of space between the two patients and the index patient. We checked the movements of these two patients and found no possible indirect transmission via contact with the virus-shedding environment before we cleaned the GW area. Both patients were admitted to the orthopaedic department, and they and the index patient had only one thing in common: they were cared for by the same nurse (tenth patient) or same resident and intern (tenth and eleventh patient). Further retrospective analysis verified the routine nurse’s round for all patients in the same ward station and the routine rounds of the doctors who cared for orthopaedic patients on the same floor on the same evening.

Before tenth and eleventh patients were isolated, they had walked freely around all areas of the hospital; therefore, numerous instances of contacts or possible viral shedding into the environment were suspected. To protect against further transmissions by infected patients, we closed the hospital over 1 month. During this period, a total of 1019 of people were isolated; (Hospital employees were 267) 69 were isolated at the hospital, 950 were isolated at home; 220 (Hospital employees were 65) were under active daily surveillance for 3 weeks. However, there was no subsequent MERS transmission in these people (Fig. 2).

### Comparison of the contact risk and personal infection-prevention measures between the ED and GW

Table 2 shows a comparison of variables between the ED and GW groups. The ED group had a higher percentage of high-risk (14.5%) and intermediate-risk (11.8%) people than the GW group (2.8 and 0.9% respectively). People in the ED group had had more frequent direct contact with the index patient. Otherwise, in the GW only 8 people (1 patient, 1 visitor and 6 HCPs) were categorized into the high-risk group and 96.3% of people were classed as low risk. The overall attack rate was 0.4% in the ED and 1.4% in the GW. For the high-risk group, the attack was higher in the GW (16.7%; l/6) than in the ED (3%; 1/33). There was no transmission in the intermediate- and low-risk groups in the ED. Two instances of transmission were identified among the low-risk group in the GW.

**Table 2:** Comparison of the contact risk and personal infection-prevention measures between the groups stayed on emergency department (ED) and general ward (GW)

	ED (*n* = 228)	GW (*n* = 218)	*p*-value
MERS identified	1 (0.4)	3 (1.4)	0.362
Baseline			
Sex, male	108 (47.4)	101 (46.3)	0.850
Age	42 ± 20	57 ± 21	< 0.001
Immuno-compromise	7 (3.1)	18 (8.3)	0.022
Contact identified			
Touch of patient	14 (6.4)	4 (1.8)	0.028
Touch of bed or equipment	16 (7.0)	6 (2.8)	0.048
Locational risk			
High (within 2 m)	33 (14.5)	6 (2.8)	< 0.001
Intermediate (same room)	27 (11.8)	2 (0.9)	< 0.001
Low (same department)	168 (73.7)	210 (96.3)	< 0.001
Median time of stay	2 (1,5)	1 (1,1)	< 0.001
Jobs			< 0.001
Patients	68 (29.8)	109 (50.0)	
Doctor/Nurse	37 (16.2)	8 (3.7)	
Other HCP	17 (7.5)	1 (0.5)	
Visitors at same room	21 (9.2)	1 (0.5)	
Visitors at different room	85 (46.2)	99 (45.4)	
Personal protection equipment			
Protection of eye, nose and mouse			
Face shield or Goggle	0	0	–
Surgical Mask	212 (93.0)	4 (1.8)	< 0.001
Particulate respirator (N95)	2 ()	0	–
Gloves			
Surgical Glove	3 (1.3)	1 (0.5)	0.624
Gown/Coverall			
Disposable gowning	0	0	–
Disposable coverall	0	0	–
Foot wear			
Waterproof boots	0	0	–
Head protection			
Head cover	0	0	–
Hand hygiene			
Hand washing	228 (100%)^a^	218 (100%)^a^	–
Hand-rubbing observed in CCTV	218 (95.6)	Not checkable	–
Post-identification measure			
Isolated	228 (100%)	8 (3.7%)	
Non-isolated and active surveillance		210 (96.3%)	

*MERS* Middle East respiratory syndrome, *CCTV* the closed-circuit television

^a^ All recalled that they washed their hands, but the time and place were unclear

Analysis of PPE usage showed a higher rate of surgical mask wearing in the primary interest group in the ED than in the GW (93.0% vs. 1.8%). In the interviews, near all people recalled that they washed their hands during a stay in the ED or GW stay, but the time and place were unclear. Some did not remember exactly and some were dropped from the closed-loop interview process, and we could not obtain correct data for hand washing in the study population. However, by reviewing CCTV, we identified obvious hand-rubbing using portable hand sanitizer (hydroalcoholic antiseptic gel for skin and hands) for 218 of the 228 (95.6%) people during their time in the ED stay. Wearing gloves was confirmed for all HCPs who had been in contact with the index patient during their caring process including changing bedding or bag, blood or urine sampling, physical examination and ECG checking. During ED and GW stays, the index patient had no complaints of respiratory symptoms and there were no aerosol-generating procedures (lung aspiration, suctioning, intubation or bronchoscopy) before the initiation of isolation. After isolation began, two doctors and nine nurses cared for index patient routinely wearing level D equipment (N95 mask, head cover, goggles, surgical glove, disposable coverall and waterproof boots) during all aerosolizing procedures.

## Discussion

This outbreak of MERS in South Korea had entirely nosocomial, human-to-human and hospital-to-hospital transmission patterns. The outbreak was initiated by a failure to identify an imported emerging infectious disease and was then amplified through the unique health care system in South Korea [13]. South Korea has a nationwide health insurance system, which controls overall medical costs en bloc and provides a flexible medical service-delivery system without barriers between small and large hospitals [11, 13]. All Koreans can be treated at a low cost and can easily visit an ED in a tertiary hospital without a referral. To reduce costs, hospital rooms generally have multiple beds per room, as did the ED in our tertiary hospital. This system provides convenient medical service to patients at low cost, but it can be vulnerable to transmission of infectious diseases between hospitals. Early in the MERS outbreak in South Korea, some infected patients visited the ED of a tertiary hospital, but this hospital failed to recognize that the patient had MERS because of delayed information for the transmission tree from the national health care ministry and rapid movement of the patient to another hospital before the recognition of MERS infection at the previous hospital [16].

MERS patients may seek a medical facility early because of serious illness, but they may expose others if the infection is not identified quickly. In the Middle East, unrecognized cases are frequently reported and are related to a sudden peak of nosocomial infection [3, 4, 17]. In South Korea, lack of awareness about this emerging infection and experience with serious outbreaks of infection may have contributed to the low compliance of HCPs for maintaining PPE and hand hygiene in the hospital [11, 13]. Therefore, the unrecognized MERS patients were easily able to transmit the infection to others during their stay in hospital. In the South Korea outbreak, a total of 186 confirmed cases were reported, 153 (83.2%) of which had derived from 5 super-spreading events [16]. In these cases, transmission occurred mainly before these patients were isolated. Two of the super-spreading events were responsible for many cases of MERS transmission in the ED space in Hospital A (2nd super-spreading), Hospital B and our hospital (index patient in this study). All 3 hospitals were located in Seoul with a population of ≈10 million (Fig. 1). The long hospital stays and crowding in these EDs contributed strongly to the higher risk of coming into contact with infected patients or exposure to infected droplets.

A study of the outbreak in the ED of Hospital A initiated from the 2nd super-spreading event revealed a high potential risk of multiple transmissions of MERS from an unrecognized patient in a crowded ED [14]. This study showed that tracing of the contact with this patient was important for predicting the attack rate of nosocomial MERS infection. Because of the close exposure of other patients, there was a high risk of contact or exposure to droplets around this patient, which may have contributed to the high attack rate. In that study, people who stayed in the same zone in the ED (close contact area) had a 20% attack rate (23 of 117). By contrast, the attack rates were only 5% for others who stayed in different zones (3 of 58) despite a time overlap with the MERS patient in the ED before that patient was isolated and 1% (4 of 500) in those with no time overlap. This study emphasizes the importance of an isolation and surveillance strategy based on contact tracing.

The epidemiological results from our hospital ED contrast with the transmission results from Hospital A. In contrast to the high transmission rate from the 2nd super-spreading event in the ED of Hospital A, in our hospital, only 1 person, who worked in the radiology suit at ED, was confirmed with MERS. Although the index patient had a mild fever (37.5 °C) and lack of respiratory symptoms, we assumed that the index patient had a sufficient risk of transmission of MERS to others when this patient visited our ED. Five infected cases were identified at another hospital, and 2 ambulance workers were confirmed as having acquired MERS during transportation of the index patient before this patient visited our ED. In our post hoc comparison between the ED and GW, the ED group had a higher overall risk of MERS than the GW group. The overlap time with the index patient was much longer in the ED than in the GW (8 h vs. 1 h), and a higher percentage (23.1%) were classified in the high-risk group in the ED group (within 2 m of the index patient). However, the overall occurrence rate was only 0.6% in the ED group and there was no transmission among the 60 people (42.0%) who stayed in the same area of the ED, including the high-risk area within 2 m of the index patient. Otherwise GW showed relative higher transmission rate than ED. One of 8 people (16.7%) who stayed in the same room as the index patient in the GW was identified as having MERS. Surprisingly, two transmissions occurred in the low-risk group (staying on the same floor but no contact), which had been thought unlikely to be infected. These epidemiological features seemed to contradict previous knowledge of MERS transmission.

Our investigation has unique characteristics. The index patient could not walk by herself because of the femur fracture, which allowed us to obtain clear information about patient’s movement and contacts during the stay. Early acquisition of all available videos and assessment of closed-looped interviews can provide useful information for the investigation of outbreaks in hospitals. Using these, we were able to obtain comprehensive information to investigate the direct and indirect transmission risks, including possible virus shedding into the environment. We also obtained objective data by observation of PPE and hand hygiene of hospital staff at the same time.

Based on these data, we propose three issues for consideration when developing a MERS transmission and prevention policy. First, a useful assumption from our analysis is that human-to-human transmission can occur even with a very brief exposure time. Generally, a shorter duration of contact time may reduce the risk of transmission because of the lower chance of contact or exposure to droplets [6, 7]. However, more cautious measures should be considered because the duration of exposure to our index patient was very brief. In our case review, all four MERS patients who were in contact with or within 2 m of the index patients had a very brief contact time (all < 5 min). The short duration did not translate to a lower risk of MERS transmission in this case. This suggests that MERS transmission can occur by brief human-to-human contact more easily than has been thought.

Second, we identified a possible pattern of human-to-human MERS transmission via the hands of medical employees who were in contact with an infected patient. In our post-hoc analysis of the tracing process, we missed two case of MERS infected form the index patient (tenth and eleventh patient). Although these two patients were admitted on the same floor, there was no exposure to the index patient. Considering the long incubation period of over 14 days, both patients may have been infected from a contaminated environment on this floor after MERS patient was isolated. However, this is seldom a possible route of action because of the following facts. When we identified index patient, we cleaned all environments of hospital. The following day, we began checking daily for presence of the MERS virus for several days and confirmed no existence of the MERS virus. The transmission routes are not easily identified under current knowledge about contact or droplet transmission or indirect transmission via a contaminated environment. An attending doctor or nurse came into contact with the index patient during the physical examination or patient care and examined other admitted patients, including touching those patients, on the same day. The possible explainable route was transmission through the hands of medical employees who had touched the index patient’s body or the virus-shedding environment. Unfortunately, we could not perform a serological evaluation of samples from the hands of the HCPs and we could not confirm this transmission route. However, we conclude that transmission via medical employees’ hands or body was the most likely explanation for MERS transmission in these patients.

Conventionally, isolation of people in response to a MERS outbreak is mainly initiated by identifying the epidemiological contact history [18]. However, considering the possibility of indirect transmission via virus shedding into the environment [19, 20], some researchers have suggested that people who share space and time with a patient should also be included in the quarantine group despite the lack of direct contact. During the early phase of the MERS outbreak in South Korea, quarantine was mainly confined to people who had come into close contact (within 2 m) with an infected person, but this has limited effects in protecting against MERS outbreaks in some hospitals [11, 12, 16]. During an outbreak, most hospitals try to isolate all people who have shared space and time with MERS patients based on this belief. However, this strategy does not include the possibility of indirect transmission via medical employees’ body or hands, which may mean that some infected people are excluded from the initial isolation process because there was no discernible contact with patients or spatial or time overlap. In contrast to the community setting, more frequent contact may occur between HCPs, patients and others in the hospital setting. Nosocomial infection may involve the transmission of a pathogen via direct contact between medical staff and other patients. We propose that during a nosocomial MERS outbreak the isolation should be extended to people who were treated by any medical employee who contacted MERS patients.

In our case, we initially missed the tenth and eleventh patients during the first isolation phase because they did not made contact with the index patient and had not shared time and space with the index patient. This had a disastrous outcome. They went around everywhere in the hospital before they were isolated. As a result, numerous people were exposed to the tenth and eleventh MERS infected patients or shared space and time with MERS patients. Although we thought that other people may seldom become infected because of the early isolation of infected patients, we should have performed extensive quarantine procedures for all people in the hospital because of the national fear of MERS transmission and the urgency of abating MERS transmission. Fortunately, among the 1019 people, there were no transmission cases. We believed that following concrete infection control measures may greatly contribute to preventing further transmission of MERS. Daily monitoring can detect these patients early and minimize the risk of MERS transmission. In-hospital campaigns of basic infection control for all people may be another contributing factor that prevented nosocomial infection of MERS. However, implementing unnecessarily broad isolations of hospital employees could cause a lack of adequate manpower and increased fatigue among non-isolated medical employees. During the national MERS outbreak, most hospitals suffering from nosocomial MERS transmission experienced the same, which may have resulted in the crisis of a lack of medical services in some regions. Unrecognized MERS patient often caused super-spreading events, especially in large volume hospitals, which can threaten not only a hospital system but also the regional medical service system.

Our study confirmed the importance of a routine basic infection policy for blocking widespread propagation of nosocomial MERS infection. Our post hoc analysis identified cases involving close contact over the long duration (8 h) of the index patient in the ED. Considering the previous report of an unrecognized case in ED [14, 17], our infection control team predicted widespread nosocomial infection at our ED. However, the outcome turned out to be quite different from what we had anticipated. During the analysis, all researchers agreed that our routine basic infection-prevention policy in the ED may have greatly contributed to the lack of MERS transmission in the ED space. Being aware of the potential for unrecognized MERS patients to enter the ED, we strengthened the initial triage in the ED to select patients at risk of MERS and encouraged all involved in the ED, including HCPs, patients and visitors, to routinely wear a surgical mask and to wash their hands frequently. Although we failed to isolate the index patient at the initial triage, high compliance with routine surgical mask wearing and hand hygiene was observed in nearly all people in the ED. In particular, we placed many hand sanitizers near beds and stations in the ED to allow all HCPs and visitors to wash their hands easily at any time. The CCTV review showed that > 95% of people in the ED washed their hands using the portable hand sanitizers. The high compliance rate of surgical mask wearing may have contributed to prevention of direct transmission of droplets into the respiratory tract and from hand to mouth or nose [21].

As overseas travel increases, the spread of emerging infections such as MERS by returning travellers is a major concern for many countries. Although the risk of encountering a patient with an emerging infection in the ED is slight, lack of recognition of emerging infections may cause serious problems for regional and national health care systems. Transmission may be extended to include hospital-to-hospital transmission, and a MERS outbreak can pose a risk to the national health care system.

Many studies have reported on the heterogenetic epidemic results of nosocomial MERS infection. Some hospitals have experienced serious nosocomial outbreaks [3, 4, 14, 17], but others [7–10, 22], mainly outside the Middle East, have reported a lack of MERS in hospital systems. We postulated that this phenomenon may result from complex interactions between the strong transmission via human-to-human contact and infection-prevention measures in each medical institution.

## Conclusions

Our analysis shows that MERS can be easily transmitted by diverse routes in the hospital setting. Routine infection-prevention practices, such as wearing a surgical mask and hand hygiene, can reduce the risk of nosocomial infection. Routine infection-prevention policies should be established in all medical institutions during a MERS outbreak. Routine precautions may play an essential role in early protection against transmission. To provide wider protection against infection, specific isolation strategies should be considered in the case of a nosocomial outbreak of MERS.

## Data Availability

The datasets generated and analyzed during the current study are not publicly available due to informations that could potentially identify individuals, but anonymous datasets are available from the corresponding author on reasonable request.

## Abbreviations

CCTV: Closed-circuit television
ED: Emergency department
GW: General wards
HCP: Healthcare provider
MERS: Middle East respiratory syndrome coronavirus
PPE: Personal protection equipment
